# Comparative efficacy of anthropometric indices in predicting 10-year ASCVD risk: insights from NHANES data

**DOI:** 10.3389/fcvm.2024.1341476

**Published:** 2024-02-29

**Authors:** Li Tang, Ling Zeng

**Affiliations:** ^1^ Department of Critical Care Medicine, West China Hospital, Sichuan University, Chengdu, China; ^2^ West China School of Nursing, Sichuan University, Chengdu, China

**Keywords:** anthropometric indices of obesity, 10-year ASCVD risk, NHANES (National Health and Nutrition Examination Survey), a body shape index (ABSI), United States

## Abstract

**Background:**

Cardiovascular diseases remain a leading cause of morbidity and mortality worldwide. Accurately predicting the 10-year risk of Atherosclerotic Cardiovascular Disease (ASCVD) is crucial for timely intervention and management. This study aimed to evaluate the predictive performance of six anthropometric indices in assessing the 10-year ASCVD risk.

**Methods:**

Utilizing data from the National Health and Nutrition Examination Survey (NHANES) database (1999–2018), the study involved 11,863 participants after applying exclusion criteria. Six anthropometric indices—waist circumference (WC), body mass index (BMI), waist-to-height ratio (WHtR), a body shape index (ABSI), body roundness index (BRI), and waist-to-height^0.5^ ratio (WHT.5R)—were calculated. The 10-year ASCVD risk was assessed using the 2013 ACC/AHA guidelines & pooled cohort equations model. Participants were divided into two groups based on an ASCVD risk threshold of 7.5%. Statistical analysis included chi-square tests, odds ratios, and receiver operating characteristic (ROC) curves.

**Results:**

The study found significant differences in baseline characteristics between participants with ASCVD risk less than 7.5% and those with a risk greater than or equal to 7.5%, stratified by gender. In both male and female groups, individuals with higher ASCVD risk exhibited higher age, waist circumference, BMI, and a higher prevalence of health-compromising behaviors. ABSI emerged as the most accurate predictor of ASCVD risk, with the highest area under the curve (AUC) values in both genders. The optimal cut-off values for ABSI was established for effective risk stratification (cut-off value = 0.08).

**Conclusion:**

The study underscores the importance of anthropometric indices, particularly ABSI, in predicting the 10-year risk of ASCVD. These findings suggest that ABSI, along with other indices, can be instrumental in identifying individuals at higher risk for ASCVD, thereby aiding in early intervention and prevention strategies.

## 1 Introduction

Atherosclerotic cardiovascular diseases (ASCVD), encompassing stroke, myocardial infarction, and sudden cardiac death (1), consume a significant portion of healthcare budgets and represent a substantial financial burden globally (2). Despite a notable reduction in cardiovascular disease mortality over the past decades, ASCVD remains the primary preventable cause of death and continues to have a high incidence in both developed and developing nations, including sub-Saharan Africa (3). In the United States, ASCVD is the leading cause of death, incurring an estimated annual medical cost exceeding $200 billion (4). This high cost is largely attributed to ineffective implementation of prevention strategies and the prevalence of uncontrolled risk factors among a considerable number of adults. Identifying the high-risk ASCVD population is crucial for primary prevention. In 2013, the American College of Cardiology/American Heart Association (ACC/AHA) introduced the Pooled Cohort Equations (PCE) to estimate the 10-year risk of a first ASCVD event. These equations have been widely endorsed by numerous guidelines as a reliable tool for assessing the 10-year risk of ASCVD. The initial risk scoring by PCE for ASCVD is also critically important. Furthermore, PCE has been recommended in hypertension guidelines to guide pharmacotherapy (5).

Obesity, a significant risk factor for ASCVD, can be classified into general obesity, typically measured by body mass index (BMI), and central adiposity, often evaluated using waist circumference (WC) or waist-to-height ratio (WHtR) (6, 7). These indices, however, do not differentiate between subcutaneous and visceral fat, the latter being more closely linked to metabolic disorders and the incidence of ASCVD (8). Advanced imaging methods like Computed Tomography (CT) and Magnetic Resonance Imaging (MRI) offer more precise measurements of visceral fat but are costly and require specialized equipment (9). In contrast, recent biomarkers such as the visceral adiposity index (VAI) and lipid accumulation product (LAP) also necessitate invasive methods for accurate measurement (10, 11). Hence, there is a need for simpler, non-invasive techniques to assess abdominal obesity. The body roundness index (BRI) and a body shape index (ABSI) have emerged as new indices of body geometry and are effective predictors of visceral fat (12, 13). These new anthropometric indices are based on a geometric model of the human body, reflecting visceral adipose tissue in a three-dimensional context. Additionally, the new waist-to-height^0.5^ ratio (WHT.5R), rooted in the power law of allometric growth, exhibits a stronger correlation with abdominal visceral fat mass and metabolic disease risk compared to previous indices (14, 15).

Prior studies have shown varying results regarding the effectiveness of different anthropometric measures in predicting ASCVD risks, emphasizing the necessity for a comprehensive analysis and comparison of these indices. This study aims to address this need by comparing the predictive power of six anthropometric indices—WC, BMI, WHtR, ABSI, BRI, and WHT.5R—in assessing the 10-year risk of ASCVD. Leveraging extensive NHANES data, which encompasses a broad demographic and provides a significant sample size for robust statistical analysis, this research is vital in determining the most effective anthropometric indices for ASCVD risk prediction, potentially guiding clinical practices and public health policies towards early intervention and prevention of cardiovascular diseases.

## 2 Materials and methods

### 2.1 Study population

This study was conducted using data from the NHANES database, covering the years 1999 to 2018. NHANES is a program of studies designed to assess the health and nutritional status of adults and children in the United States, which is conducted by the National Center for Health Statistics (NCHS) of the Centers for Disease Control and Prevention (CDC). This database provides a comprehensive overview of the health and nutritional status of the general population and includes demographic, socioeconomic, dietary, and health-related information.

**Figure 1:**
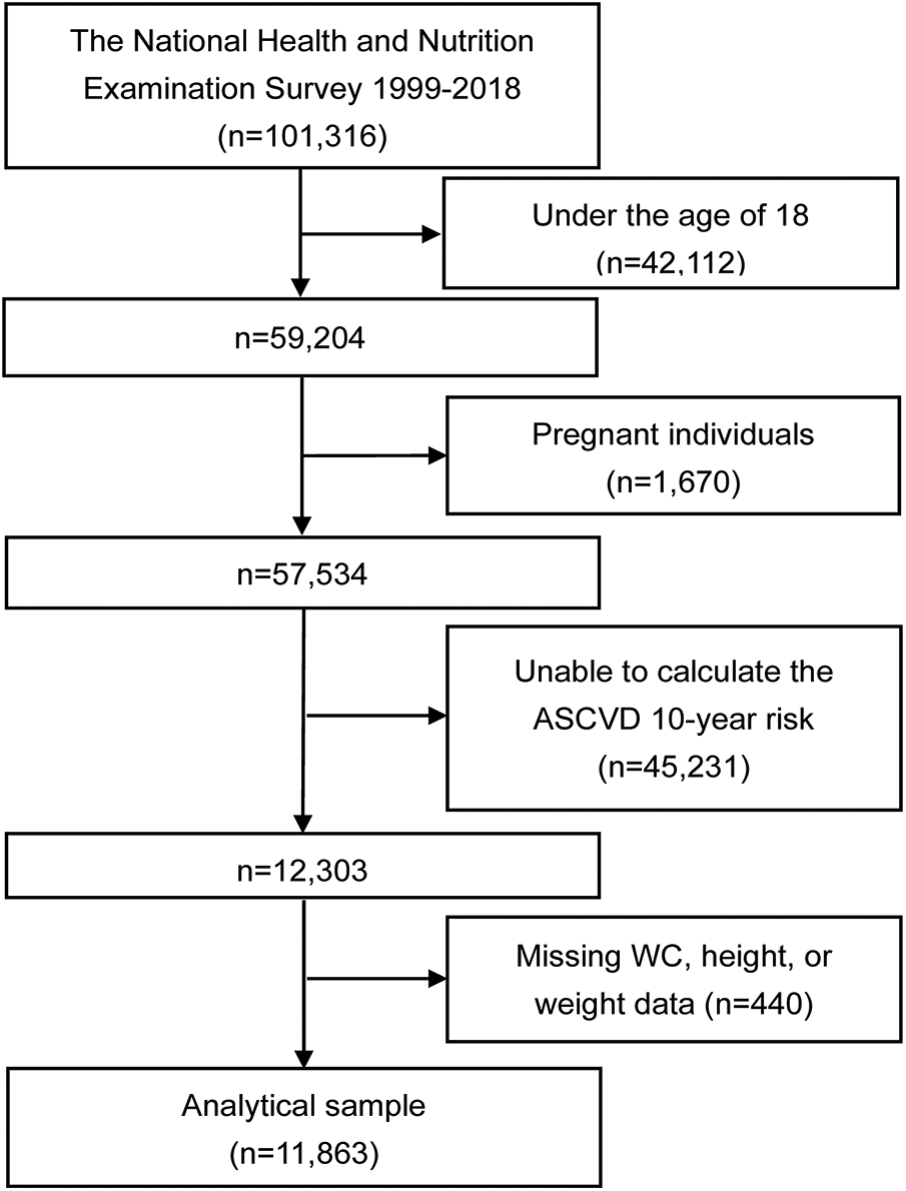
Flowchart of the study. ASCVD, atherosclerotic cardiovascular disease; WC, waist circumference.

From an initial pool of 101,316 participants, the study sample was refined through a series of exclusion criteria. Individuals under the age of 18 (42,112 cases) were first excluded. Pregnant participants (1,670 cases) were also excluded due to the unique physiological changes that occur during pregnancy, which could affect the study's variables. Participants for whom the ASCVD 10-year risk could not be calculated (45,231 cases) and those with missing key variables such as waist circumference, height, or weight (440 cases) were also excluded from the analysis. After applying these criteria, the final study sample consisted of 11,863 participants (Figure 1).

### 2.2 Calculation of six anthropometric indices

The study focused on six anthropometric indices: WC, BMI, WHtR, ABSI, BRI, and WHT.5R. The specific algorithms for calculating these indices are as follows:

(1) WC: Measured in centimeters (cm) around the smallest area below the rib cage and above the belly button.
(2) $$\mathrm{BMI}=\frac{\mathrm{weight}\;\left(\mathrm{kg}\right)}{\mathrm{height}\;{\left(\mathrm{cm}\right)}^{2}}$$
(3) $$\mathrm{WHtR}=\frac{\mathrm{WC}\;\left(\mathrm{cm}\right)}{\mathrm{height}\;\left(\mathrm{cm}\right)}$$
(4) $$\mathrm{ABSI}=\frac{\mathrm{WC}\;\left(m\right)}{\mathrm{BMI}\;{\left(\mathrm{kg}/m^{2}\right)}^{\frac{2}{3}}\times \mathrm{height}\;{\left(m\right)}^{\frac{1}{2}}}$$
(5) $$\mathrm{BRI}=364.2-365.5\times \sqrt{1-\left(\frac{{\left(\mathrm{WC}\;\left(m\right)/\left(2\pi \right)\right)}^{2}}{{\left(0.5\times \mathrm{height}\;\left(m\right)\right)}^{2}}\right)}$$
(6) $$\mathrm{WHR}.5R=\frac{\mathrm{WC}\;\left(\mathrm{cm}\right)}{\sqrt{\mathrm{height}\;\left(\mathrm{cm}\right)}}$$

### 2.3 Calculation of ASCVD 10-year risk

The 10-year risk of ASCVD was calculated using the PCEs model as per the 2013 ACC/AHA guidelines (1). This model incorporates demographic factors (age, gender), blood cholesterol levels, blood pressure, smoking status, and diabetes history to estimate the likelihood of experiencing a first severe ASCVD event within the next decade. A threshold of 7.5% was used to categorize the 10-year ASCVD risk, with values equal to or greater than this threshold considered indicative of high risk (16).

### 2.4 Other variables

In addition to the primary variables of interest, the study also collected and analyzed data on demographic variables, lifestyle factors, anthropometric measurements, and laboratory tests. This information was gathered through computer-assisted personal interviews (CAPI) during NHANES data collection. Demographic variables included age, gender, poverty income ratio, and education level. Lifestyle factors encompassed smoking, alcohol consumption, physical activity, and medication history. Physical examinations were performed to gather data on waist circumference, blood pressure, height, and weight.

The poverty income ratio (PIR) was categorized as low (below 1.3), medium (1.3–3.5), and high (above 3.5). Smoking status was categorized into three groups: never smokers (individuals who have smoked fewer than 100 cigarettes in their lifetime), former smokers (those who have smoked more than 100 cigarettes but have ceased smoking at the time of the survey), and current smokers (individuals who have smoked more than 100 cigarettes and continue to do so). Similarly, current drinking status was classified into three tiers: heavy drinking (for females, this means consuming 3 or more drinks per day or engaging in binge drinking on 5 or more days per month; for males, it entails consuming 4 or more drinks per day or the same frequency of binge drinking), moderate drinking (for females, this includes consuming 2 or more drinks per day or binge drinking on at least 2 days per month; for males, it involves consuming 3 or more drinks per day or the same frequency of binge drinking), and mild drinking (encompassing all other cases). Physical activity was assessed based on the metabolic equivalent of task (MET) per week, a metric derived by multiplying the total minutes spent on various activities during the week by their respective metabolic equivalents, as outlined in the Compendium of Physical Activities. This physical activity was further segmented into three categories: low (less than 600 METs/week), moderate (600–1,199 METs/week), and vigorous (1,200 METs/week or more). Laboratory tests included the estimation of the glomerular filtration rate (eGFR) using the 2009 serum creatinine (SCr) Chronic Kidney Disease Epidemiology Collaboration (CKD-EPI) equation (17).

### 2.5 Statistical analysis

Data analysis was performed using R software (version 3.5.3) and EmpowerStats. We presented continuous variables as either mean ± standard deviation or median (interquartile range), contingent on their distribution patterns. Proportions were used to represent qualitative data. The analysis of categorical variables was carried out using the chi-square test. The study calculated odds ratios (OR) with 95% confidence intervals (CI) to examine the correlations between six anthropometric indices and the 10-year ASCVD risk. Notably, to address dimensional differences, a *Z*-score transformation was applied to the six anthropometric indices in regression analysis. In addition, the discriminative capacity of these indices was assessed via the receiver operating characteristic (ROC) curve. The indices' cutoff values were established based on the maximum Youden index in the ROC curve. DeLong's method was utilized to analyze the significance of the area under the curve (AUC) when comparing different anthropometric indices (18). A *P*-value below 0.05 was deemed indicative of statistical significance.

## 3 Results

### 3.1 Baseline characteristics

Table 1 presents the foundational characteristics of the participants, differentiated by ASCVD 10-year risk (less than 7.5% and greater than or equal to 7.5%), and further stratified by gender. The study comprised 6,040 male and 5,823 female participants. In the male cohort, no significant disparity in weight (*P* = 0.472) was observed between the two risk groups. However, males in the higher risk group (ASCVD ≥ 7.5%) displayed characteristics such as advanced age, increased WC and BMI, elevated systolic blood pressure, decreased diastolic blood pressure, lower educational and economic status, and a higher prevalence of current and former smoking, as well as former alcohol consumption. Additionally, this group exhibited reduced physical activity, lower eGFR, and a higher frequency of antidiabetic, lipid-lowering, and antihypertensive medication use. The female group mirrored these trends, with similar baseline characteristic differences between the two ASCVD risk categories.

**Table 1:** Baseline characteristics of subjects.

Characteristics	Male				Female			
	Total *n* = 6,040	10-year risk of ASCVD <7.5% *n* = 2,279	10-year risk of ASCVD ≥7.5% *n* = 3,761	*P*-value	Total *n* = 5,823	10-year risk of ASCVD <7.5% *n* = 3,647	10-year risk of ASCVD ≥7.5% *n* = 2,176	*P*-value
Age (years)	58.92 ± 11.39	48.33 ± 5.80	65.34 ± 8.87	<0.001	58.49 ± 11.25	52.05 ± 7.71	69.29 ± 7.26	<0.001
WC (cm)	105.95 ± 14.45	102.59 ± 14.31	107.98 ± 14.15	<0.001	98.07 ± 16.08	96.22 ± 16.46	101.17 ± 14.91	<0.001
Height (cm)	176.24 ± 7.07	178.06 ± 6.90	175.13 ± 6.95	<0.001	162.28 ± 6.44	163.42 ± 6.29	160.36 ± 6.24	<0.001
Weight (kg)	90.84 ± 18.92	91.06 ± 18.91	90.70 ± 18.92	0.472	76.78 ± 19.09	76.98 ± 19.82	76.46 ± 17.81	0.317
BMI (kg/m^2^)	29.20 ± 5.63	28.68 ± 5.52	29.52 ± 5.67	<0.001	29.16 ± 7.08	28.83 ± 7.31	29.71 ± 6.64	<0.001
WHtR	0.60 ± 0.08	0.58 ± 0.08	0.62 ± 0.08	<0.001	0.61 ± 0.10	0.59 ± 0.10	0.63 ± 0.09	<0.001
ABSI	0.085 ± 0.004	0.082 ± 0.004	0.086 ± 0.004	<0.001	0.082 ± 0.005	0.081 ± 0.005	0.084 ± 0.005	<0.001
BRI	5.60 ± 1.97	5.03 ± 1.85	5.95 ± 1.96	<0.001	5.75 ± 2.43	5.40 ± 2.43	6.33 ± 2.31	<0.001
WHT.5R	7.98 ± 1.08	7.69 ± 1.06	8.16 ± 1.05	<0.001	7.70 ± 1.27	7.53 ± 1.29	7.99 ± 1.17	<0.001
SBP (mmHg)	126.33 ± 16.92	119.77 ± 13.07	130.31 ± 17.73	<0.001	125.83 ± 19.23	119.22 ± 15.16	136.92 ± 20.19	<0.001
DBP (mmHg)	72.74 ± 11.57	75.22 ± 10.14	71.23 ± 12.12	<0.001	70.66 ± 11.31	72.16 ± 9.96	68.11 ± 12.90	<0.001
Education level (%)				<0.001				<0.001
Less than high school	16.64	10.45	20.39		14.80	11.00	21.15	
High school	25.55	23.53	26.77		27.38	23.30	34.21	
More than high school	57.81	66.02	52.83		57.83	65.70	44.64	
PIR (%)				<0.001				<0.001
Low	20.37	18.19	21.72		22.35	19.65	27.04	
Medium	33.35	25.85	37.96		34.88	28.52	45.88	
High	46.27	55.96	40.32		42.77	51.83	27.09	
Smoking (%)				<0.001				0.010
Never	37.25	50.46	29.25		51.14	52.67	48.58	
Former	41.14	32.21	46.56		28.44	27.61	29.83	
Now	21.61	17.33	24.20		20.42	19.71	21.60	
Drinking (%)				<0.001				<0.001
Never	5.03	4.21	5.53		13.09	9.71	18.75	
Former	20.18	14.04	23.90	<0.001	18.17	14.45	24.40	
Mild	44.60	44.98	44.38	<0.001	34.09	33.92	34.38	
Moderate	9.82	12.59	8.14	<0.001	19.03	23.14	12.13	
Heavy	15.56	20.01	12.87		10.24	13.49	4.78	
Not reported	4.80	4.17	5.18		5.39	5.29	5.56	
METs/week (%)				<0.001				<0.001
Low	25.12	27.12	23.90		28.35	30.00	25.60	
Moderate	2.75	3.25	2.45		2.46	2.36	2.62	
Vigorous	52.17	56.74	49.40		42.02	45.90	35.52	
Not reported	19.97	12.90	24.25		27.17	21.74	36.26	
eGFR (ml/min/1.73 m^2^)	82.39 ± 17.39	90.75 ± 13.73	77.32 ± 17.41	<0.001	82.58 ± 18.42	88.64 ± 15.71	72.40 ± 18.13	<0.001
Antidiabetic medications (%)	12.53	3.42	18.05	<0.001	9.36	4.20	18.01	<0.001
Lipid lowering medications (%)	31.37	16.37	40.47	<0.001	24.11	15.03	39.34	<0.001
Antihypertensive medications (%)	41.44	19.13	54.96	<0.001	40.63	25.99	65.17	<0.001

ASCVD, atherosclerotic cardiovascular disease; WC, waist circumference; BMI, body mass index; WHtR, waist-to-height ratio; ABSI, a body shape index; BRI, body roundness index; WHT.5R, waist/height^0.5^; SBP, systolic blood pressure; DBP, diastolic blood pressure; PIR, poverty income ratio; MET, metabolic equivalent of task; eGFR, estimated glomerular filtration rate.

### 3.2 Association between anthropometric indices and 10-year ASCVD risk

As depicted in Table 2, the study analyzed the correlation between six anthropometric indices and the 10-year risk of ASCVD. In the initial unadjusted model (Model 1), a rise in ASCVD risk was linked with increased values of anthropometric measurements. In a more comprehensive, multivariable-adjusted model (Model 3), which accounted for potential confounders like age, gender, education level, PIR, smoking and alcohol habits, physical activity (measured in METs), eGFR, and medication use (antidiabetic, lipid-lowering, and antihypertensive), the association between these anthropometric indices and ASCVD risk remained statistically significant. This relationship was consistent in both the overall population and within gender-specific subgroup analyses.

**Table 2:** ORs and 95% CIs for 10-year high risk of ASCVD of different anthropometric indexes (*Z*-score) in male and female.

	Total	Male	Female
Model 1			
WC	1.60 (1.54, 1.67)	1.57 (1.47, 1.67)	1.37 (1.30, 1.45)
BMI	1.15 (1.10, 1.19)	1.21 (1.13, 1.29)	1.13 (1.07, 1.18)
WHtR	1.58 (1.52, 1.65)	1.92 (1.79, 2.06)	1.51 (1.43, 1.59)
ABSI	2.88 (2.73, 3.03)	4.05 (3.69, 4.44)	2.05 (1.93, 2.19)
BRI	1.51 (1.45, 1.58)	1.88 (1.75, 2.02)	1.44 (1.37, 1.52)
WHT.5R	1.61 (1.55, 1.68)	1.74 (1.62, 1.86)	1.44 (1.36, 1.52)
Model 2			
WC	1.71 (1.61, 1.83)	1.68 (1.53, 1.84)	1.77 (1.61, 1.93)
BMI	1.51 (1.41, 1.60)	1.57 (1.43, 1.73)	1.47 (1.35, 1.59)
WHtR	1.82 (1.71, 1.95)	1.89 (1.71, 2.09)	1.79 (1.64, 1.96)
ABSI	1.74 (1.62, 1.87)	1.96 (1.73, 2.21)	1.65 (1.50, 1.81)
BRI	1.74 (1.64, 1.86)	1.83 (1.66, 2.02)	1.70 (1.56, 1.85)
WHT.5R	1.77 (1.66, 1.89)	1.78 (1.62, 1.96)	1.78 (1.63, 1.94)
Model 3			
WC	1.73 (1.59, 1.88)	1.91 (1.69, 2.16)	1.59 (1.40, 1.79)
BMI	1.61 (1.48, 1.75)	1.97 (1.73, 2.24)	1.40 (1.25, 1.58)
WHtR	1.79 (1.64, 1.95)	2.13 (1.87, 2.43)	1.58 (1.40, 1.78)
ABSI	1.27 (1.17, 1.39)	1.33 (1.15, 1.54)	1.24 (1.10, 1.39)
BRI	1.66 (1.53, 1.81)	1.99 (1.75, 2.27)	1.47 (1.31, 1.65)
WHT.5R	1.76 (1.62, 1.92)	2.02 (1.78, 2.30)	1.58 (1.40, 1.78)

Model 1: Non-adjusted.

Model 2: Adjusted for age, sex.

Model 3: Adjusted for age, sex, education level, PIR, smoking, drinking, MET, eGFR, antidiabetic medication, lipid lowering medication, and antihypertensive medication.

The sex variables were not adjusted in the stratified analysis of sex.

OR, odds ratio; CI, confidence interval; ASCVD, atherosclerotic cardiovascular disease; WC, waist circumference; BMI, body mass index; WHtR, waist-to-height ratio; ABSI, a body shape index; BRI, body roundness index; WHT.5R, waist/height^0.5^; PIR, poverty income ratio; MET, metabolic equivalent of task; eGFR, estimated glomerular filtration rate.

### 3.3 AUCs and cut-off values for anthropometric indices in ASCVD risk prediction

**Figure 2:**
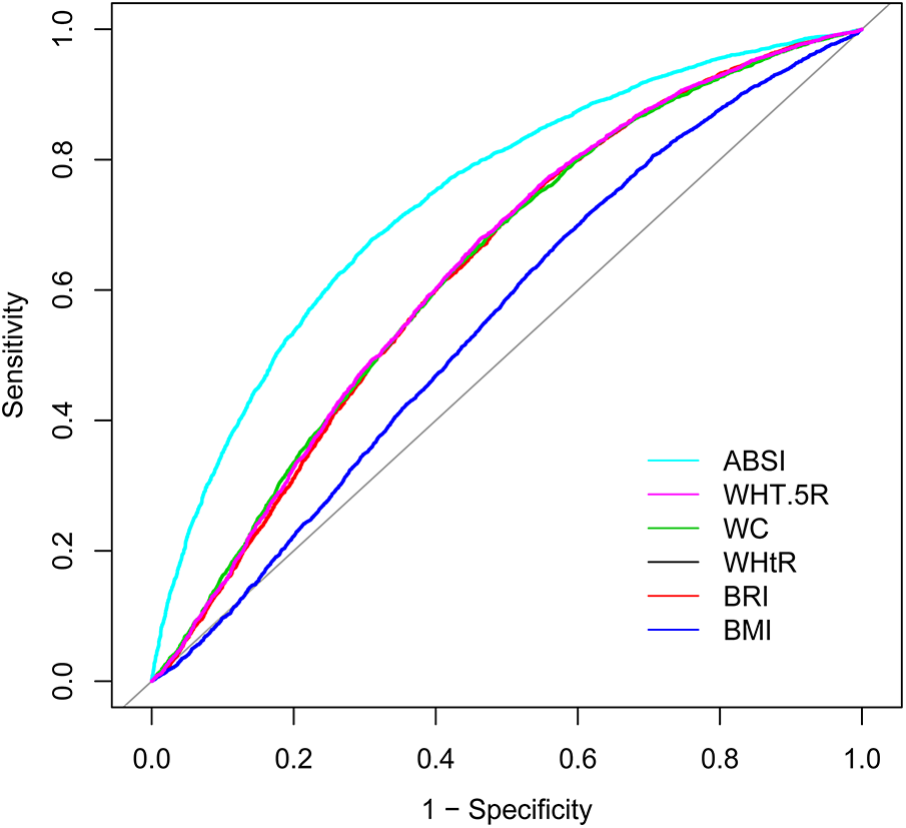
ROC of different anthropometric indexes for predicting 10-year high risk of ASCVD. ABSI, a body shape index; WHT.5R, waist/height^0.5^; WC, waist circumference; WHtR, waist-to-height ratio; BRI, body roundness index; BMI, body mass index; ROC, receiver operating characteristic curve; ASCVD, atherosclerotic cardiovascular disease.

**Figure 3:**
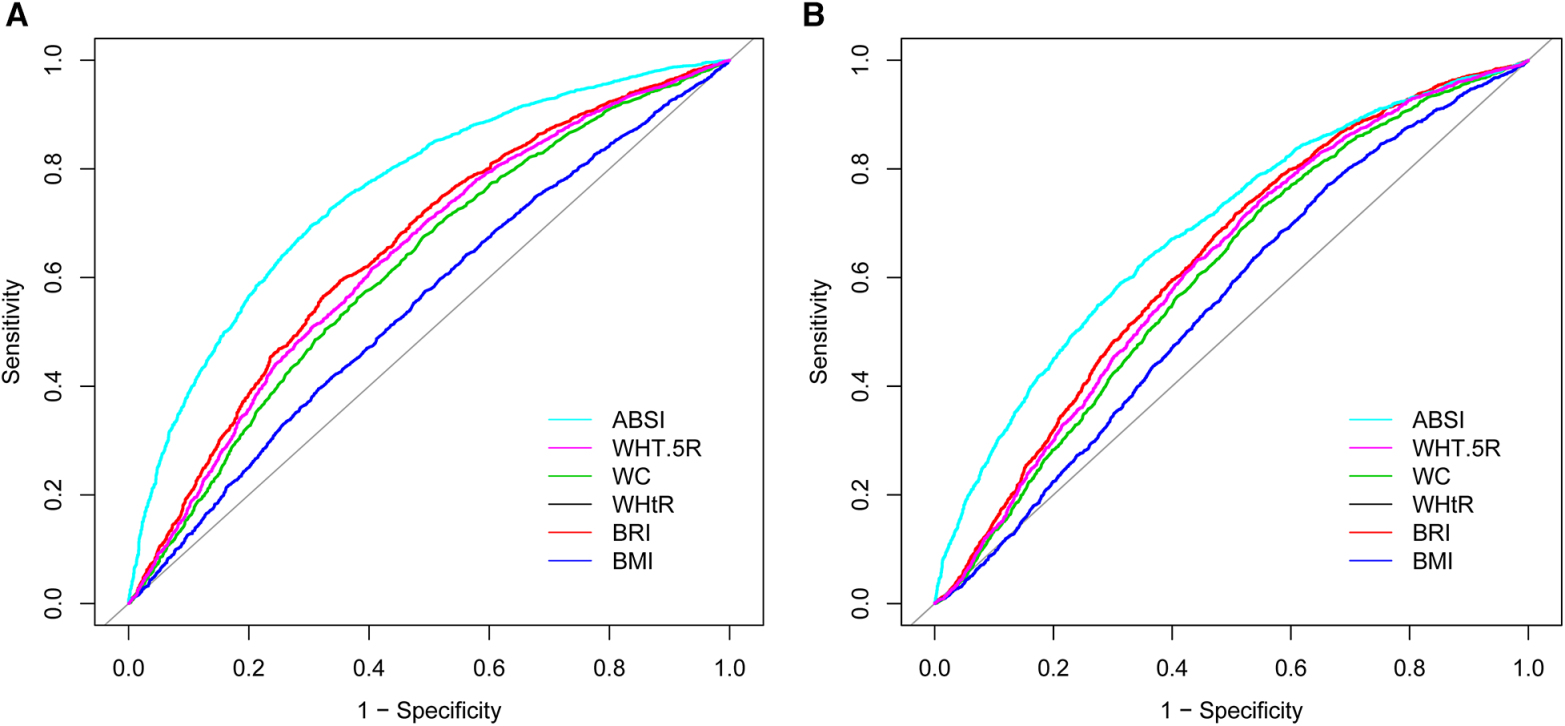
ROCs of different anthropometric indexes for predicting 10-year high risk of ASCVD in (**A**) male and in (**B**) female. ABSI, a body shape index; WHT.5R, waist/height^0.5^; WC, waist circumference; WHtR, waist-to-height ratio; BRI, body roundness index; BMI, body mass index; ROC, receiver operating characteristic curve; ASCVD, atherosclerotic cardiovascular disease.

Table 3, alongside Figures 2, 3, elucidates the predictive efficacy of the six anthropometric indices in determining ASCVD risk, as measured by the AUC. The ABSI exhibited the highest AUC value (AUC = 0.74), followed by the WHT.5R with an AUC of 0.64. The derived optimal cutoff values for ABSI and WHT.5R, based on their specificity and sensitivity, are 0.08 and 7.57, respectively (Figure 2). In predicting ASCVD risk among males, ABSI retained superior accuracy (AUC = 0.76) compared to other indices, as demonstrated in Figure 3A, followed by the WHtR and BRI, both achieving an AUC of 0.65. The corresponding optimal cutoffs for ABSI, WHtR, and BRI are established at 0.08, 0.59, and 5.27, respectively. Similar trends were observed in the female cohort, with ABSI, WHtR, and BRI leading in AUC values (Figure 3B). In summary, the evaluation of six anthropometric indicators revealed that ABSI had the strongest predictive ability for assessing the 10-year risk of ASCVD in both male and female cohorts, followed by WHtR and BRI, then WHT.5R and WC, with BMI demonstrating the least predictive capacity.

**Table 3:** AUC and cut-off values of different anthropometric indexes for predicting 10-year high risk of ASCVD.

Variables	AUC (95% CI)	Cut-off	Specificity	Sensitivity
Total				
WC	0.63 (0.62–0.64)	98.35	0.53	0.68
BMI	0.55 (0.54–0.56)	26.06	0.39	0.71
WHtR	0.63 (0.62–0.64)	0.57	0.49	0.72
ABSI	0.74 (0.73–0.75)	0.08	0.69	0.68
BRI	0.63 (0.62–0.64)	4.78	0.49	0.72
WHT.5R	0.64 (0.63–0.65)	7.57	0.54	0.68
Male				
WC	0.62 (0.60–0.63)	101.15	0.51	0.67
BMI	0.55 (0.54–0.57)	27.91	0.51	0.57
WHtR	0.65 (0.64–0.67)	0.59	0.65	0.59
ABSI	0.76 (0.74–0.77)	0.08	0.70	0.69
BRI	0.65 (0.64–0.67)	5.27	0.65	0.59
WHT.5R	0.64 (0.62–0.65)	7.79	0.60	0.62
Female				
WC	0.60 (0.59–0.62)	91.85	0.45	0.73
BMI	0.55 (0.54–0.57)	24.70	0.34	0.77
WHtR	0.63 (0.62–0.65)	0.58	0.49	0.72
ABSI	0.69 (0.67–0.70)	0.08	0.65	0.63
BRI	0.63 (0.62–0.65)	4.88	0.49	0.72
WHT.5R	0.62 (0.60–0.63)	7.17	0.44	0.76

AUC, area under the curve; ASCVD, atherosclerotic cardiovascular disease; CI, confidence interval; WC, waist circumference; BMI, body mass index; WHtR, waist-to-height ratio; ABSI, a body shape index; BRI, body roundness index; WHT.5R, waist/height^0.5^.

## 4 Discussion

This research assessed the predictive capability of six anthropometric indices in determining the 10-year risk of ASCVD, offering valuable perspectives for ASCVD risk evaluation. The findings indicate that individuals with a heightened risk of ASCVD tend to exhibit higher average anthropometric measurements, applicable to both males and females. In the context of multivariate adjusted logistic regression, all the anthropometric values demonstrated a statistical correlation with ASCVD risk. The analysis using the ROC curve identified ABSI as the most efficient predictor of ASCVD risk, marked by the highest AUC value for both genders.

The research by Agbo et al. suggests that, for populations in sub-Saharan Africa, abdominal height may be a more accurate measure for assessing cardiovascular risk than BMI and WHR (19). This conclusion is drawn from the premise that cardiovascular diseases are more closely linked to abdominal fat, particularly around visceral organs, rather than to subcutaneous fat (20). Measurements like abdominal height or sagittal abdominal diameter could reflect visceral obesity levels in a completely non-invasive manner. In contrast, Wu et al.'s study suggests that for assessing the 10-year high risk of cardiovascular disease in males, the waist-to-hip ratio (WHR) is the most effective anthropometric method, whereas the WHtR and BRI are the best for women. In contrast, the ABSI was found to have poor predictive ability in both male and female populations (21). This variation could be due to differences in adipose tissue distribution among races and the specific characteristics of their study's target population (22), which consisted of individuals from hospital health examinations typically maintaining better health and lifestyle habits. Wang et al.'s research, aligning more closely with our findings, identified ABSI as the best anthropometric indicator for assessing coronary heart disease risk in men, proposing a cutoff value of 0.078 (23). This study also noted gender differences in the predictive power of anthropometric indices for coronary heart disease, with WHtR and BRI emerging as the most effective for women, a point also emphasized in Wu et al.'s study (21). In our research, while ABSI was the top predictor for ASCVD risk, WHtR and BRI were secondary but more effective than WC and BMI. This suggests that ABSI, originally developed based on the American population (13), might have limited applicability in other demographic groups. Despite ABSI's advantage in avoiding collinearity issues seen in other anthropometric indices, caution is advised when applying it as a routine measurement outside the United States.

Furthermore, our study, like Wang et al.'s, found that various anthropometric indices exhibit similar predictive powers across genders. Although previous studies indicate that sex hormones influence fat formation, with a propensity for men to accumulate more visceral adipose tissue (24), and emphasize different anthropometric indices for predicting gender-specific cardiovascular disease risks (21, 25, 26), ABSI's comprehensive approach—encompassing height, weight, and WC—may offer a more accurate assessment of obesity in different genders. This is because ABSI accounts for both the linear relationship between WC and BMI and the nonlinear relationship between WC and height (13, 27), potentially making it a more precise indicator of central obesity and body fat distribution across genders.

Fat deposition is notably linked to an elevated risk of both incidence and mortality rates in cardiovascular diseases (28). Apart from BMI, all other anthropometric measures in our study can be utilized to assess fat deposition in the abdominal cavity. Notably, aside from ABSI, which demonstrated the highest predictive ability for ASCVD risk, WHtR and BRI showed strong predictive capabilities in both male and female populations. This might be attributed to the fact that both BRI and WHtR calculations are based on WC and height. Supporting this, a meta-analysis by Paajanen et al. revealed that shorter adults have a higher incidence of cardiovascular diseases compared to taller individuals, with the shortest adults facing a 50% higher risk and mortality from coronary heart disease (29). Moreover, Henriksson et al.'s study found a negative correlation between the height of middle-aged men and their serum cholesterol levels, including non-high-density lipoprotein cholesterol, a relationship independent of BMI (30). This finding offers partial insight into why WHtR and BRI outperform BMI and WC in ASCVD risk assessment. Further expanding on the evaluation of anthropometric indices, Liu et al.'s study identifies WHtR as a simple and effective measure for evaluating cardiovascular metabolic risk factors in non-obese adults, suggesting BRI as a viable alternative (31). This finding highlights the similar predictive performance of these two measures. Additionally, WHT.5R, which seems to be a modification of the WHtR formula (altering the power of the denominator) (32), displayed relatively strong predictive performance in ROC analysis comparisons.

It is notable that traditional measures such as BMI and waist circumference, while still relevant, may not be as predictive of cardiovascular risk as these newer indices. This finding aligns with those of previous studies. Specifically, Ashwell et al.'s meta-analysis revealed that compared to BMI, WC marginally improves the detection rate of adverse outcomes by 3%, while the WHtR enhances this rate by 4%–5%. Notably, WHtR is substantially more effective than both WC and BMI in predicting diabetes, hypertension, CVD, and overall outcomes for both genders (7). Furthermore, Adegbija et al. observed that WC and BMI have comparable predictive capacities for estimating the absolute risk of cardiovascular disease (33). This underscores the need for a more nuanced approach to obesity and cardiovascular risk assessment, moving beyond general obesity to consider the distribution and type of adipose tissue. This issue suggests the need for more comprehensive or alternative measurement methods, like the ABSI, which encapsulates the interrelationships between height, weight, and WC. ABSI thus offers a more nuanced and reliable assessment of cardiovascular and metabolic risk factors, potentially improving the accuracy of health risk predictions.

The design of the ABSI specifically targets a more accurate portrayal of the interplay between waist circumference and body shape, particularly focusing on the health consequences of abdominal obesity (13). In contrast with BMI, ABSI reduces the influence of weight and height, thereby emphasizing the role of waist circumference. Since weight and height are incorporated within the BMI, which cannot completely differentiate between muscle and fat or accurately reflect fat distribution (34–36), waist circumference becomes a more significant measure of central obesity—a key factor associated with cardiovascular disease risk (37). In the ABSI algorithm, the inclusion of weight in the denominator is intended to reduce the effect of body weight on waist circumference, while the adjustment of height's proportion aims to lessen the relative impact of an individual's height on waist circumference. These methodological adjustments could influence the sensitivity and specificity of ASCVD risk predictions in individuals with varying body measurements.

Moreover, the current study has observed a diminished OR for ABSI in predicting ASCVD risk. This could be attributed to ABSI's extensive range, which in turn, may yield a compressed OR in logistic regression analyses. To counteract the dimensional disparities among six anthropometric indices, *Z*-score have been employed within a logistic regression framework to standardize these measures. Nevertheless, ABSI's *Z*-score exhibit the greatest range (with respective ranges for WC, BMI, WHtR, ABSI, BRI, and WHT.5R at 6.9, 7.87, 6.58, 11.48, 7.44, and 6.52), potentially diminishing the OR in logistic regression. Furthermore, ABSI may share a more pronounced correlation with variables adjusted in the logistic regression analysis, such as demographic and lifestyle factors, which could be an additional factor in the attenuated OR value. It is critical to elucidate that a significant effect size of a biomarker within logistic regression implies a robust direct correlation with ASCVD. However, this is not inherently indicative of its ultimate predictive capacity to distinguish between affected and unaffected cohorts. Hence, the relatively modest OR values associated with ABSI in logistic regression should not be construed as detracting from the overall conclusions of our study, but rather as indicative of a nuanced relationship with ASCVD risk factors that merits further examination for more effective risk assessment and screening modalities.

Our findings have important implications for clinical practice and public health strategies. The identification of ABSI as a strong predictor of ASCVD risk suggests that this measure could be incorporated into routine clinical assessments to improve risk stratification and guide preventive interventions. Additionally, public health campaigns could benefit from emphasizing the importance of monitoring central obesity and its implications for cardiovascular health. While our study provides valuable insights, it is not without limitations. The cross-sectional nature of the NHANES database limits the ability to establish causality. Furthermore, the applicability of our findings may be restricted due to the particular demographic and health characteristics of the NHANES cohort and the scope of the PCE formula. Future studies are encouraged to broaden the scope of research to include diverse populations, such as those in sub-Saharan Africa and China (38, 39), utilizing cardiovascular risk formulas tailored to these groups to address the current limitations of extrapolation associated with the PCE formula. Additionally, there is a need for longitudinal research to validate our findings and further investigate the mechanisms linking anthropometric indicators with ASCVD risk.

## 5 Conclusion

In conclusion, this study contributes to the growing body of evidence supporting the use of novel anthropometric indices, particularly ABSI, in cardiovascular risk assessment. These findings highlight the importance of considering various aspects of body composition in predicting ASCVD risk and underscore the potential of these measures in improving cardiovascular health outcomes.

## Data Availability

The raw data supporting the conclusions of this article will be made available by the authors, without undue reservation.
